# Cosmeceutical and Wound-Healing Activities of Green Hydroxypropyl-*β*-Cyclodextrin-Glycerol-Based *Satureja montana* Extracts

**DOI:** 10.3390/molecules30122638

**Published:** 2025-06-18

**Authors:** Lejsa Jakupović, Jakub W. Strawa, Laura Nižić Nodilo, Marijan Marijan, Anita Hafner, Katarzyna Jakimiuk, Monika Tomczykowa, Michał Tomczyk, Marijana Zovko Končić

**Affiliations:** 1 Department of Pharmacognosy, University of Zagreb Faculty of Pharmacy and Biochemistry, A. Kovačića 1, 10000 Zagreb, Croatia; lejsa.jakupovic@pharma.unizg.hr (L.J.); u3ne86@gmail.com (M.M.); 2 Department of Pharmacognosy, Faculty of Pharmacy with the Division of Laboratory Medicine, Medical University of Białystok, ul. Mickiewicza 2a, 15-230 Białystok, Poland; jakub.strawa@umb.edu.pl (J.W.S.); katarzyna.jakimiuk@umb.edu.pl (K.J.); michal.tomczyk@umb.edu.pl (M.T.); 3 Department of Pharmaceutical Technology, University of Zagreb Faculty of Pharmacy and Biochemistry, A. Kovačića 1, 10000 Zagreb, Croatia; laura.nizic@pharma.unizg.hr (L.N.N.); anita.hafner@pharma.unizg.hr (A.H.); 4 Department of Organic Chemistry, Faculty of Medicine with the Division of Dentistry and Division of Medical Education in English, Medical University of Białystok, ul. Mickiewicza 2a, 15-222 Białystok, Poland; monika.tomczyk@umb.edu.pl

**Keywords:** *Satureja montana*, anti-inflammatory, antioxidant, biocompatibility, cyclodextrins, cosmeceutical, tyrosinase, wound healing

## Abstract

*Satureja montana* L. (winter savory, family Lamiaceae) is an aromatic herb that is widespread throughout the Mediterranean region. In a prior study, the optimization of the green hydroxypropyl-*β*-cyclodextrin (HP-*β*-CD)-glycerol-assisted extraction procedure of *S. montana* was performed. As a result, four extracts abundant in total phenols (OPT-TP), total phenolic acids including rosmarinic acid (OPT-TPA-RA), total flavonoids (OPT-TF), and luteolin derivatives (OPT-LG) showing anti-elastase and anti-hyaluronidase properties, were prepared. Subsequently, we further explored the phytochemical, dermatological, and cosmeceutical potentials of these extracts, evaluating their antioxidant, anti-inflammatory, anti-tyrosinase, and anti-ultraviolet (UV) absorption activities. Furthermore, the biocompatibility of the extracts and their wound-healing properties were assessed using HaCaT cells. The results indicate that the extracts exhibited excellent antioxidant and cosmeceutical activities, which surpassed the activities of the employed standards in several assays (DPPH antiradical activity, *β*-carotene-linoleic acid, anti-lipoxygenase, anti-heat-induced ovalbumin coagulation, and UV absorbance assays). Furthermore, the extracts preserved more than 80% of the HaCaT cell viability at concentrations up to 62.5 µL extract/mL and also enhanced wound healing in the in vitro scratch wound-healing model. For example, the application of OPT-TP and OPT-TF led to 48.6% ± 3.3% and 48.6% ± 5.4% wound closure, respectively, after 48 h, compared to 34.8% ± 2.3% in the control group. The extracts exhibited excellent bioactivities, making them promising candidates for the development of cosmeceutical products, while their high biocompatibility indicates that they are suitable for direct application in cosmetics without prior solvent removal.

## 1. Introduction

With the increasing significance of health and physical appearance, the cosmetic industry is actively seeking innovative strategies to meet the evolving needs and expectations of consumers. In pursuit of this objective, the term “cosmeceuticals” has emerged to characterize products that are believed to possess both cosmetic and medicinal properties. These products can be viewed as functionalized cosmetics that provide therapeutic benefits in addition to their aesthetic purposes. Although the word “cosmeceutical” lacks a formal legal definition, it is frequently utilized by both consumers and industry professionals, as it bridges the gap between cosmetic and pharmaceutical products [1]. Plant extracts, due to their positive effects on skin health and the consumer preference for products with natural ingredients, are the most promising potential ingredients of cosmeceutical products [2]. Numerous studies have demonstrated that they can function as antioxidants or protect the skin’s macromolecules from enzymatic degradation associated with aging and environmental exposure [3].

The first step for the incorporation of bioactive plant metabolites into cosmetic formulations is their effective extraction from plant sources. As modern consumers grow more conscious of environmental concerns, there is a heightened demand for the “green” extraction of bioactive natural products from plant materials. Such eco-friendly and sustainable methods use green solvents that are non-flammable and safe for both human health and the environment [4,5]. Glycerol is one of the best examples of a green solvent due to its natural origin, environmentally friendly characteristics, affordability, and safety. Furthermore, as a by-product of the biodiesel manufacturing process, glycerol has the added advantage of being produced from sustainable and renewable sources [6]. Finally, glycerol has the additional advantage of being skin-friendly and is thus widely utilized as a frequent ingredient of cosmetic products, where it functions as a humectant, moisturizer, and viscosity-regulating agent [4]. Another example of green solvents are aqueous solutions of cyclodextrins (CDs): non-toxic, environmentally friendly cyclic oligosaccharides with six (*α*-CD), seven (*β*-CD), or eight (*γ*-CD) d-glucopyranoside subunits in a ring. The exterior of CDs is hydrophilic due to the presence of hydroxyl groups on the glucose units, while the interior cavity is hydrophobic due to the orientation of the glycosidic bonds. This structural configuration allows CDs to form inclusion complexes with various small molecules, thereby enhancing their solubility and stability in water [7]. CDs in cosmetic formulations have the additional benefits of improving bioavailability [8] and facilitating the transport of active compounds across the epidermal barrier [9].

The addition of polar side chains can further improve the aqueous solubility and stability of CDs and their complexes. One of the more well-known examples is (2-hydroxypropyl)-*β*-cyclodextrin (HP-*β*-CD), *β*-CD modified by an extra hydroxypropyl group. HP-*β*-CD is often used to increase the solubility of flavonoids, as illustrated by the encapsulation of rutin within HP-*β*-CD complexes [8] or kaempferol derivatives [10]. As both HP-*β*-CD and glycerol have high biocompatibility, it is possible to integrate them into various cosmetic formulations. This means that they do not need to be removed from the final extract, thereby saving time and costs and reducing the CO_2_ footprint from production. Consequently, the use of HP-*β*-CD-glycerol ultrasound-assisted extraction (HCDGUAE) for obtaining bioactive compounds from plant sources presents a highly attractive green alternative to conventional extraction. Despite their excellent safety and extraction efficiencies, the combination of HP-*β*-CD with glycerol has rarely been used for the extraction of natural products. Among the few examples are the extraction of polyphenols from oak acorn husks [11], olive leaves [12], and *Helichrysum italicum* [13,14].

Winter savory (*Satureja montana* L., Lamiaceae) is an aromatic plant that is widespread throughout the Mediterranean region and is rich in various phenolic components, of which the most represented are rosmarinic acid (RA) and other caffeic acid derivatives. Another large group of phytochemicals found in *S. montana* is the flavonoids, the majority of which are derivatives of luteolin and apigenin. However, derivatives of quercetin and catechin may also be present [15,16]. Additionally, *S. montana* contains triterpenic acids such as ursolic or oleanolic acid, as well as essential oil rich in carvacrol (53.35%) [17], and it exhibits a range of properties relevant to skin health and appearance. Both *S. montana* extracts and its essential oil demonstrate significant efficacy against various dermatopathological micro-organisms, including *Trichophyton violaceum*, *T. rubrum*, *T. tonsurans*, and *T. mentagrophytes* [18]. Furthermore, alcoholic *S. montana* extract exhibited good in vitro anti-inflammatory activity, such as the inhibition of cyclooxygenases (COXs), superoxide dismutase, and catalase, as well as a good total antioxidant capacity [19]. Polyphenolics of *S. montana*, such as RA and luteolin derivatives, also display numerous skin-related activities. Luteolin derivatives accelerate wound healing and act as anti-inflammatory, antioxidant, anti-collagenase, and anti-hyaluronidase agents [20], while RA is one of the most researched phytochemicals for skincare due to its antimicrobial, immunomodulatory, anti-allergic, and anti-inflammatory properties [21].

In our previous study [22], an exhaustive optimization of the HCDGUAE of *S. montana* was performed. As a result, four extracts with the maximal contents of total phenols (OPT-TP), total phenolic acids including RA (OPT-TPA-RA), total flavonoids (OPT-TF), and luteolin derivatives (OPT-LG) were prepared. The extracts presented potent anti-elastase and anti-hyaluronidase properties, indicating a significant cosmeceutical potential. In this study, this potential of OPT-TP, OPT-TPA-RA, OPT-TF, and OPT-LG was further investigated. The anti-melanogenic, UV-protective, anti-inflammatory, biocompatibility, and wound-healing properties of the prepared extracts were investigated to assess their efficacy and suitability for direct application in cosmetic formulations. In addition, the detailed phytochemical compositions of the four extracts were determined using LC-MS analysis.

## 2. Results and Discussion

### 2.1. LC-MS Analysis of the Extracts

Plant polyphenolics are commonly used in cosmetic formulations due to their diverse biological properties that contribute to anti-aging, including antioxidant capabilities, dermal protease inhibition, and protection against ultraviolet (UV) radiation [23]. Flavonoids are polyphenolic compounds ubiquitous in the plant kingdom, and their chemical structure, which is rich in conjugated double bonds, is responsible for their excellent antioxidant properties, which help protect both the skin and cosmetic formulations from harmful free radicals and UV radiation [24]. In addition, flavonoids can act as anti-melanogenic agents [25]. Other groups of polyphenolics such as phenolic acids also display strong antioxidant properties, as well as anti-inflammatory, photoprotective, and depigmenting effects. Furthermore, they have the potential to inhibit the activities of matrix metalloproteinases, including collagenase, and thereby function as anti-aging ingredients in cosmetic products [26].

Previous studies on various extracts and preparations have shown that *S. montana* is a rich source of diverse phenolic compounds [16], and the results of the chromatographic analysis presented herein confirm that the HCDGUAE method developed in previous works is well suited for their extraction. UV chromatograms of the prepared extracts recorded at 280 nm and 350 nm reveal the presence of thirteen compounds (Figure S1). Detailed information on the identified compounds is available in Table 1 and Figures S2–S14, where the compounds are listed in order of elution from the column and numbered accordingly. The same numbering system is maintained throughout the text.

After the interpretation of the fragmentation patterns from the collected mass spectra of the extracts, the general structures of the phenolic compounds were tentatively identified via a comparison with the literature and/or confirmed via an exact mass match and isotopic fragment pattern comparison with the employed standard. Of the former, eight compounds were identified as flavonoid compounds, mostly belonging to luteolin derivatives (**1**, **3**, **5**–**7**), while apigenin and quercetin derivatives were represented with one compound each (**2** and **4**, respectively). The aglycone of one remaining flavonoid could not be identified by the means used in this method. In addition, caffeic acid derivatives were represented with five compounds.

In the previous study using HPLC-PDA analysis [22], we tentatively identified and reported the presence of RA and luteolin 7-*O*-glucoside. In this experiment, the initial LC-MS/TOF analysis confirmed the presence of RA, while the presence of luteolin 7-*O*-glucoside was questioned. Our LC-MS analysis, conducted under different selected chromatographic conditions and correlated with the retention time, the UV spectrum, and a comparison of the obtained MS mass spectra with the standard, confirmed that the luteolin derivative observed in the previous studies was, in fact, luteolin 7-*O*-glucuronide. The current results largely concur with those of a study investigating the compositions and antimicrobial activities of decoctions of *S. montana* from Portugal. That study reported the luteolin derivative abundance, as well as the presence of large amounts of RA and other caffeic acid derivatives, including lithospermic and salvianolic acid derivatives [27], recorded in this study. Similar results were obtained when investigating the chemical composition of the *Satureja montana* subsp. *kitaibelii* from Serbia [28]. In addition to RA and luteolin, other phytochemicals present in *S. montana* extracts may exert positive effects on the skin. For example, both lithospermic [29] and salvianolic [30] acid restored the skin barrier functions in the imiquimod-induced psoriasis-like animal model.

### 2.2. Antioxidant Activities of S. montana Extracts

Cosmetic products often contain compounds prone to oxidative degradation, such as polyunsaturated fatty acids, the properties of which should be maintained during their storage. During their use and application, the product’s ingredients are exposed to atmospheric oxygen; thus, it is essential to incorporate free radical scavengers or reducing agents to their formulations, such as vitamins and plant extracts [31]. The added antioxidants can also actively protect dermal macromolecules from oxidative harm inflicted on the skin by environmental elements, including UV radiation and free radicals [32,33]. To determine the antioxidant properties of the *S. montana* extracts, three in vitro methods were used: The 2,2-diphenyl-1-picrylhydrazyl (DPPH)-radical scavenging activity, reducing power, and *β*-carotene-linoleic acid assay methods. The activities of the extracts were compared to those of standard antioxidants, namely, butylated hydroxyanisole (BHA) and ascorbic acid (ASC). In the assays performed in this study, the IC_50_ of the extracts and standards are expressed in different measurement units (μL extract/mL and μg/mL, respectively). The reason is that, unlike the standards, which are solid substances easily dissolved in the desired concentration, the extracts could not be evaporated to dryness as glycerol, the co-solvent present in the extracts, is an involatile substance. Thus, it was not possible to directly compare the activities of the standards and the extracts. However, it is possible to regard the numerical values of IC_50_ of the standards as volume (in μL) equivalents of 1 mg/mL solutions. With this in mind, the activities of the standard antioxidants in the performed assays were measured and reported for general comparison purposes.

**Figure 1:**
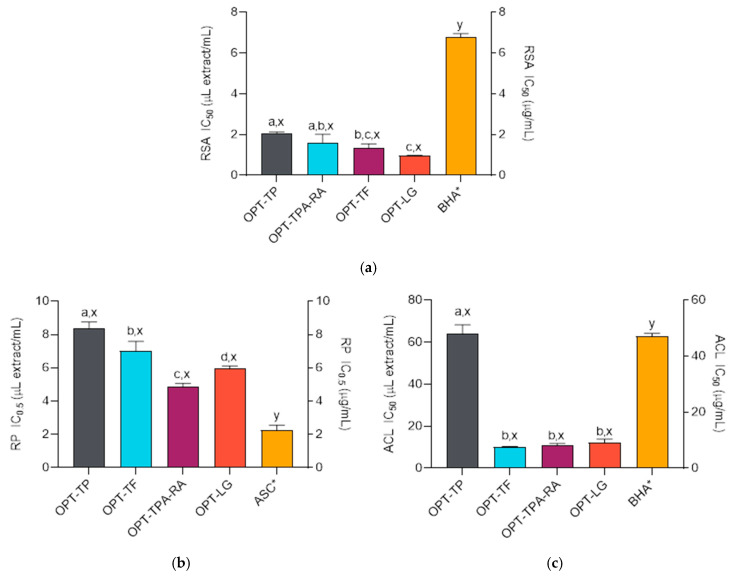
Radical scavenging activities (RSA) (**a**), reducing powers (RP) (**b**), and activities in the *β*-carotene-linoleic acid assay (ACL) (**c**) of *S. montana* extracts (prepared as described in Table 2) and the positive controls, BHA (butylated hydroxyanisole) and ASC (ascorbic acid). ^a,b,c,d^ Differences between the extracts (ANOVA followed by Tukey’s post-test, *p* < 0.05). ^x,y^ Differences from the positive control (ANOVA followed by Dunnett’s post-test, *p* < 0.05). Columns not connected with the same letters are statistically different. The asterisk indicates that the result is plotted on the right ordinate. The details of the IC_50_ and IC_0.5_ values calculations are given in the respective subsections of the Section 3.

The antioxidant activity of *S. montana* essential oil has also been investigated in several studies [34,35], while the extract activities have been less researched [36]. The studies have shown the excellent antioxidant properties of both the volatile and non-volatile substances. The results of the antioxidant assays performed in this work demonstrate that the HCDGUAE method was effective at preserving the antioxidant properties of the plant. The extracts showed considerable activities in all the performed assays, but these activities depended on the assay (Figure 1a–c). For example, the extracts displayed excellent reducing power, albeit lower than that of the ASC. However, the extracts displayed outstanding radical scavenging activities (RSAs), which surpassed those of the standard antioxidant BHA. Similarly, the extracts also displayed excellent activities in the *β*-carotene-linoleic acid assay. While OPT-TP was somewhat less active than BHA, the other extracts were better inhibitors of carotene bleaching than the employed standard (Dunnett’s post-test, *p* < 0.05). The group of compounds responsible for RSA are probably the total phenolic compounds, as there was a statistically significant correlation between the TP and RSA (*r*^2^ = 0.9382, *p* < 0.05). OPT-TF and OPT-LG were among the most active extracts in all three assays. The strong antiradical and reducing powers of these extracts might be related to the presence of luteolin derivatives and other flavonoids. The structural features of luteolin enable it to donate hydrogen electrons and stabilize the radical species [37]. Caffeic acid and its derivatives also have strong antioxidant properties, sometimes surpassing those of well-established antioxidants, such as Trolox or ASC [38].

### 2.3. Photoprotective and Anti-Melanogenic Potentials of the S. montana Extracts

**Figure 2:**
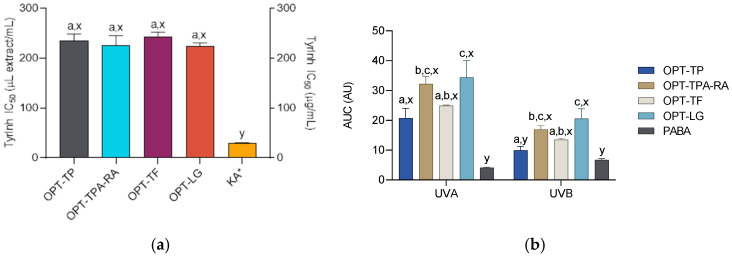
Tyrosinase inhibitory activity (TyInh) (**a**) and areas under curves (AUC) for UV spectra in the UVA and UVB regions (**b**) of the *S. montana* extracts (prepared as described in Table 2) and the positive controls KA (kojic acid) and 1 mg/mL *p*-aminobenzoic acid (PABA) solution in a 1:64 dilution. ^a,b,c^ Differences between the extracts (ANOVA followed by Tukey’s post-test, *p* < 0.05). ^x,y^ Differences from the positive control (ANOVA followed by Dunnett’s post-test, *p* < 0.05). Columns not connected with the same letters are statistically different. The asterisk indicates that the dataset is plotted on the right ordinate. The details of the IC_50_ values calculations are given in the respective subsections of the Section 3.

The anti-melanogenic potential of the prepared extracts was investigated through their anti-tyrosinase and UV-absorbing properties. Tyrosinase is the most important enzyme involved in the biosynthesis of melanin, the pigment responsible for skin pigmentation and protection against UV radiation. It is a copper-dependent polyphenol oxidase that catalyzes the conversion of l-tyrosine to 3,4-dihydroxyphenylalanine (l-DOPA) and subsequently oxidizes l-DOPA to produce dopachrome, a precursor for melanin production. However, while melanin offers protective benefits for the skin, its overproduction and accumulation can result in various dermatological conditions that negatively impact the skin’s appearance, including solar lentigo, melasma, and progressive hyperpigmentation. In this context, tyrosinase inhibitors are utilized to hinder melanin synthesis, functioning as depigmenting agents in various dermatological formulations [39,40]. The activities of the extracts are presented in Figure 2a. While all the extracts exhibited tyrosinase-inhibiting activity, it was lower than that of the standard tyrosinase inhibitor kojic acid (ANOVA followed by Dunnett’s post-test, *p* < 0.05). In addition, the activity did not differ among the extracts (ANOVA followed by Tukey post-test, *p* < 0.05). Numerous plant substances may display anti-tyrosinase activity. Rosmarinic acid, one of the main phenolic compounds found in the investigated extracts, has been found to be a potent tyrosinase inhibitor [41]. Furthermore, the *S. montana* extracts were rich in different types of polyphenolics which display strong anti-tyrosinase properties [42]. However, luteolin and its derivatives, which the extracts are rich in, are not potent tyrosinase inhibitors [43], which is probably the reason for the unremarkable results that the extracts demonstrated in this assay.

Excessive or cumulative exposure to solar UV radiation may cause numerous problems related to skin health and appearance, such as uneven pigmentation, premature skin aging, dermal damage, and the potential development of skin cancers. While UV-B radiation (290–320 nm) is predominantly recognized as the primary contributor to the adverse effects of solar exposure, the detrimental impacts of UV-A radiation (320–400 nm) are also being increasingly documented [44,45]. Topical sunscreens are formulated with UV filters that protect the skin from the harmful effects of UV radiation, and the use of plant-based sunscreen alternatives is becoming increasingly popular [46]. The UV-absorbing capabilities of the examined plant extracts were assessed using the spectrophotometric method and compared to the absorbance characteristics of the topical sunscreen *p*-aminobenzoic acid (PABA). The extracts demonstrated significant UV light absorption across both the UV-B and UV-A regions (Figure 2b) that, in the majority of the extracts, was greater than that of the PABA solution, which suggests that the topical application of the investigated extracts may effectively block photons from penetrating the skin. This is likely attributable to the presence of phenolic compounds in the extracts, including luteolin and other flavonoids, which are known to absorb the full UV-B spectrum and part of the UV-A spectrum, thereby exhibiting photoprotective properties similar to those of traditional sunscreens [37,47]. Furthermore, the phytochemicals present in the extracts may offer additional benefits over traditional sunscreens. For example, in a study on human keratinocytes, RA attenuated the cell damage against UV-B radiation-induced oxidative stress by enhancing the antioxidant effects [48], while salvianolic acid B may protect against UV-B-induced skin aging via the activation of nuclear factor erythroid 2-related factor 2 (NRF2), as determined in a human dermal fibroblast model [49].

### 2.4. Anti-Inflammatory Activity of the S. montana Extracts

**Figure 3:**
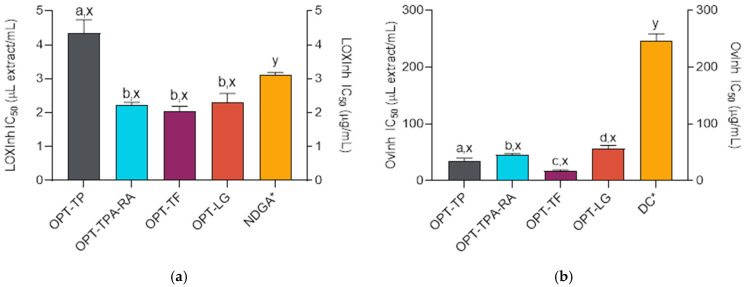
Lipoxygenase (LOX) (**a**) and ovalbumin coagulation inhibitory (OvInh) (**b**) activities of the *S. montana* extracts (prepared as described in Table 2) and the positive controls NDGA (nordihydroguaiaretic acid) and DF (diclofenac). ^a,b,c,d^ Differences between the extracts (ANOVA followed by Tukey’s post-test, *p* < 0.05). ^x,y^ Differences from the positive control (ANOVA followed by Dunnett’s post-test, *p* < 0.05). Columns not connected with the same letters are statistically different. The asterisk indicates that the dataset is plotted on the right ordinate. The details of the IC_50_ values calculations are given in the respective subsections of the Section 3.

The skin is subjected to a variety of detrimental factors that contribute to oxidative stress and compromise its integrity and homeostasis. Prolonged exposure to both exo- and endogenous reactive oxygen species can lead to chronic inflammation, accelerated skin aging, tissue damage, and immunosuppression. Many of the inflammatory skin responses, characterized by symptoms such as erythema, rashes, swelling, or impaired physiological functions, are mediated by lipoxygenase (LOX) [50]. In addition to inflammatory processes, LOX isozymes may be involved in the modulation of epithelial proliferation, wound healing, inflammatory skin diseases, and even cancer [50]. Besides their modulated enzymatic activity, another characteristic of inflammatory processes is tissue protein denaturation [51]. A well-known example is protein denaturation caused by UV radiation, which, in turn, causes the photoaging of the skin [52]. Therefore, the extracts that suppress LOX activity and protein denaturation may have cosmeceutical and anti-aging activities because they hinder the development of inflammatory skin changes and related changes in skin appearance [53]. The investigated *S. montana* extracts demonstrated excellent anti-inflammatory properties and were effective LOX inhibitors (Figure 3a). OPT-TP displayed somewhat weaker activity in this assay, but the activities of the remaining extracts were statistically equal (ANOVA followed by Tukey’s post-test, *p* < 0.05) and exceeded the activity of nordihydroguaiaretic acid (NDGA) (ANOVA followed by Dunnett’s post-test, *p* < 0.05) Furthermore, the extracts were able to impede heat-induced ovalbumin coagulation (Figure 3b) significantly better than the employed standard, diclofenac (ANOVA followed by Dunnett’s post-test, *p* < 0.05). The most active extract in this assay was OPT-TF.

Numerous phytoconstituents found in the *S. montana* extracts in this study presented strong anti-inflammatory activities, results which were not surprising. For example, lithospermic acid B [54] and luteolin were able to inhibit various isoforms of the LOX enzyme [55]. The anti-inflammatory properties of luteolin and its derivatives have also been documented in various cell types, including keratinocytes, fibroblasts, and several immune cells, such as macrophages, mast cells, neutrophils, dendritic cells, and T cells. Luteolin has the capacity to inhibit pro-inflammatory mediators, including interleukin (IL)-1*β*, IL-6, IL-8, IL-17, IL-22, tumor necrosis factor (TNF)-*α*, and COX-2, while also influencing multiple signaling pathways, such as the nuclear factor k-light-chain-enhancer of activated B cells (NF-κB) and Toll-like receptor (TLR) pathways [37]. Also, RA may add to the anti-inflammatory properties of the extracts by inhibiting LOX activity in a dose-dependent manner, as it changes the enzyme structure via hydrogen bonding and hydrophobic interaction as [56]. It seems that RA and luteolin may have a synergistic effect. A study investigating the effects of the two substances in lipopolysaccharide (LPS)-stimulated RAW264.7 macrophages found out that a combination of RA and luteolin more strongly inhibited the production of nitric oxide (NO), inducible NOS (iNOS), prostaglandin E_2_ (PGE_2_), and COX-2 than higher concentrations of RA or luteolin alone. Furthermore, the combined RA and luteolin synergistically inhibited the production of pro-inflammatory cytokines, such as TNF-*α*, IL-6, and IL-1*β*. The combination of the two phenolics suppressed NF-κB activation by inhibiting the degradation of inhibitor of NF-κB and nuclear translocation of the p65 subunit of NF-κB. The effect of the combination was stronger than the effects of RA or luteolin alone [57].

### 2.5. Influence of the S. montana Extracts on Cell Viability

**Figure 4:**
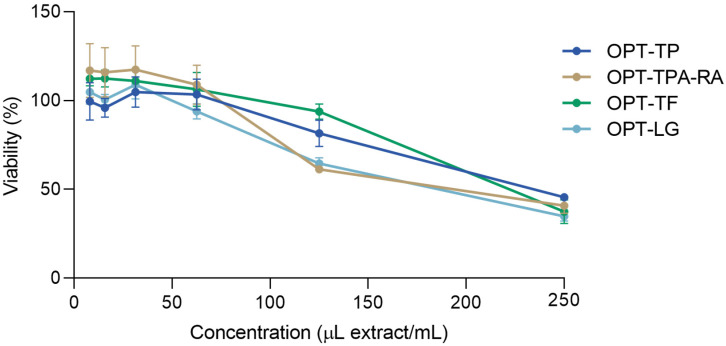
The influence of the *S. montana* extracts (prepared as described in Table 2) in different concentrations on the HaCaT cells viability. The extracts were investigated in concentrations of 250, 125, 62.5, 31.3, 15.6, and 7.8 μL extract/mL. The cell viability is expressed as a percentage compared to cells treated with HBSS designated to have 100% viability. The exact viability percentage and the statistical differences are presented in the Supplement (Table S1).

Keratinocytes account for 95% of the cells in the epidermis. Their primary function is to play the role of the structural and barrier function of the epidermis. However, their role in wound repair is also well recognized [58]. To assess the biocompatibility of the prepared *S. montana* extracts, their influence on the viability of the human aneuploid immortal keratinocyte cell line (HaCaT) was evaluated. HaCaT cells are spontaneously immortalized human keratinocytes that possess the ability to differentiate in vitro. They are considered to be a reliable in vitro differentiation model to dissect the inflammatory and repair response of human keratinocytes [58]. Different concentrations (7.8–250 μL/mL) of the extracts diluted in Hank’s balanced salt solution (HBSS) were used to estimate the toxicity of the extract HaCaT cell cultures. The findings from the 3-(4,5-dimethylthiazol-2-yl)-2,5-diphenyltetrazolium bromide (MTT) assay, illustrated in Figure 4, demonstrate that the extracts did not adversely affect the HaCaT cell viability at concentrations up to 62.5 μL/mL. In general, the activity did not statistically differ among the extracts except at the concentration of 125 μL extract/mL (Table S1). Consequently, the high viability of keratinocytes exposed to the extracts, as evidenced by this assay, suggests that these extracts possess low toxicity and are appropriate for incorporation into cosmetic formulations.

### 2.6. Wound-Healing Effects of S. montana

Wound healing encompasses a series of intricate cellular and molecular processes that unfold in several stages, which may occur concurrently: hemostasis, inflammation, proliferation/migration, and maturation or remodeling, all of which are marked by the development of new tissue. During the proliferation phase, keratinocytes and fibroblasts migrate to restore the vascular network and contribute to the granulation process, a phenomenon that is utilized in the in vitro “scratch” assay. In this method, a scratch is made on the surface of a cell monolayer placed within a well to create an empty area, or “wound”. Provided that the environmental conditions are suitable, cellular movement and proliferation ensue, leading to the gradual closure of the wound area [59].

**Figure 5:**
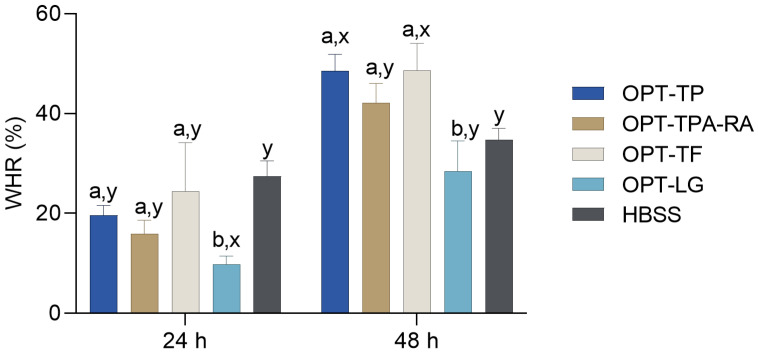
Wound-healing rates (WHRs) of *S. montana* extracts calculated as the percentage of scratch closure relative to the initial scratch area. The pixel-based measurement was performed after 24 h and 48 h. The extracts were prepared as described in Table 2. The measurement was performed after 24 h and 48 h. ^a,b^ Differences between the extracts within each time point (one-way ANOVA followed by Tukey’s post-test, *p* < 0.05). ^x,y^ Differences from the negative control within each time point (one-way ANOVA followed by Dunnett’s post-test, *p* < 0.05). Columns not connected with the same letters are statistically different.

In our research, spontaneously immortalized human keratinocyte line (HaCaT) cells were treated with the *S. montana* extracts in a concentration of 31.3 μL/mL, and HBSS was used as the negative control. For the assay, the extracts were used in a 31.3 μL/mL concentration, and their influence on the wound closure was compared with the influence of HBSS (Figure S15). The percentages of the wound healing after treatment with different samples, recorded at 24 h and 48 h, are presented in Figure 5. The wounds in the wells of the extract-treated HaCaT cells tended to be reduced over time. While the wound-healing rates of the extracts after 24 h did not statistically differ from that of the negative control (one-way ANOVA followed by Dunnett’s post-test, *p* < 0.05), those of most of the extracts surpassed that of HBSS after 48 h. Especially active were OPT-TP and OPT-TF, the extracts that accelerated wound closure in the confluent cell layer compared to HBSS (one-way ANOVA followed by Dunnet’s post-test, *p* < 0.05), indicating excellent wound-healing activity.

Luteolin and its derivatives demonstrate excellent wound-healing activity, as documented in a study on luteolin derivatives and luteolin-rich *Jasione montana* extracts on fibroblasts [60]. Furthermore, a bioassay-guided fractionation and isolation investigation of the wound-healing potential of the *Daphne oleoides* subsp. *kurdica* determined that luteolin derivatives were the main active components of the aerial parts of the plants that exerted the activity through the inhibition of hyaluronidase and collagenase activities and by interfering with the inflammatory stage [20]. A study on streptozotocin-induced diabetic rats with impaired wound healing revealed that systemic administration of luteolin improves impaired healing, accelerates re-epithelization, and ameliorates inflammation and oxidative stress. Histopathological staining and immunoblotting revealed an inhibitory effect of luteolin on inflammatory cell and cytokine production. Multiple mechanisms of luteolin activity observed in this study included decreases in protein expressions of inflammatory factors including matrix metalloproteinase (MMP)-9, TNF-α, IL-6, and IL1-β and downregulation of nuclear factor (NF)-κB, as well as increases in anti-oxidative enzymes such as superoxide dismutase (SOD)1 and glutathione peroxidase (GSH-Px) induced by NRF2 [61].

RA may also stimulate the healing process as exemplified by an experimentally induced nasal mucosal injury, most likely due to its anti-inflammatory effect [62]. In a study comparing the effects of RA and dexpanthenol in a rat experimental wound model, the authors concluded that RA can be used in topical creams for wound healing, as it leads to a significant reduction in the wound size, resulting in fewer scars [63]. Some of the RA effects may be related to the anti-inflammatory activity of RA. Namely, RA grafted dextran/gelatin hydrogel demonstrated wound healing properties in a rat model of full-thickness skin defect. The mechanism of action was proved to be related to its anti-inflammatory properties by adjusting the expression of inflammatory cytokines, such as TNF-α, and reducing the level of oxidative stress markers (malondialdehyde and hydrogen peroxide) [64]. Finally, salvianolic acids may also be excellent candidate for wound-healing preparations. Salvianolic acid B administered to fibroblasts at a concentration of 75 µg/mL enhanced cell viability and significantly promoted cell migration. Additionally, there was an observed increase in the expression of collagen type III, which plays a crucial role in the early phases of wound healing, indicating considerable potential for the application of salvianolic acid-containing preparations in wound-healing therapies [65]. The extracts prepared using HCDGUAE retained the activities of the *S. montana* constituents, yielding promising wound-healing activities.

## 3. Materials and Methods

### 3.1. Materials and Apparatus

The ASC, BHA, diclofenac, kojic acid, luteolin 7-*O*-glucuronide, MTT, NDGA, PABA, and RA were purchased from Sigma-Aldrich (St. Louis, MO, USA). The soybean LOX was obtained from TCI chemicals (Tokyo, Japan). Acetonitrile Optima was purchased from Fisher Chemical (Loughborough, UK), and the mobile phase modifier formic acid (FA) was purchased from POCH (Gliwice, Poland). The HaCaT cells were purchased from CLS Cell Line Services (Heidelberg, Germany). The buffers and chemicals used for the cell cultivation and viability experiments were as follows: HBSS (pH 6.0) (Capricorn Scientific, Ebsdorfergrund, Germany), Dulbecco’s modified Eagle’s medium (DMEM) (Sigma-Aldrich, St. Louis, MO, USA), fetal bovine serum (FBS; 10% *v*/*v* Biosera, Boussens, France), penicillin, streptomycin, and amphotericin B (5%, *v*/*v*) (Lonza, Basel, Switzerland), as well as MTT (Sigma-Aldrich, St. Louis, MO, USA). The other reagents and chemicals were analytical-grade. A SONOREX^®^ Digital 10 P DK 156 BP ultrasonic bath (Bandelin, Berlin, Germany) was used for the ultrasound-assisted extraction. The cell viability assay spectrophotometric determinations were performed using a 1420 Multilabelcounter VICTOR3 microplate reader (PerkinElmer, Waltham, MA, USA), while the measurements for the other assays were performed using the FLUOstar Omega (BMG Labtech, Ortenberg, Germany) microplate reader. A Primovert microscope (Carl Zeiss AG, Oberkochen, Germany) was used for the phase-contrast microscopy. The ultra-pure water for the LC-MS analyses was obtained in-house using a POLWATER DL3-100 deionizer (Labopol-Polwater, Kraków, Poland), and the LC-ESI-MS analyses were conducted using an Agilent Technologies 1260 Infinity chromatography system coupled to a 6230 time-of-flight mass spectrometer (TOF/MS) and PDA detector (Agilent, Santa Clara, CA, USA).

### 3.2. Plant Material

Flowering aerial parts of *S. montana* were collected in October 2022 from areas surrounding the village of Ravno (Bosnia and Herzegovina, 42°53′13″ N, 17°58′15″ E). The plant material was authenticated by Professor Antun Alegro (Faculty of Sciences, University of Zagreb, Zagreb, Croatia). A voucher specimen (SM-2022-10-1) was deposited in the plant collection of the Department of Pharmacognosy, Faculty of Pharmacy and Biochemistry, University of Zagreb (Zagreb, Croatia). Prior to extraction, the plant material was dried, reduced to a powder, and passed through a sieve with a mesh size of 850 μm.

### 3.3. Extract Preparation

Four extracts rich in total polyphenols (OPT-TP), flavonoids (OPT-TF), phenolic acids (including RA) (OPT-TPA-RA), and luteolin derivative (OPT-LG) were prepared as previously described [22]. In short, the fresh powdered plant material and HP-*β*-CD were quickly dispersed in 10 g of a water/glycerol mixture in an Erlenmeyer flask, quickly stirred, and placed in an ultrasonication bath. The detailed extraction parameters (glycerol content, HP-*β*-CD amount, temperature, ultrasonication power, and extraction time) used for the preparation of each extract are presented in Table 2. After the ultrasonication, the extracts were filtered and kept at −20 °C before use. The phytochemical compositions of the extracts, as previously reported in [22], are presented in Table 3. However, the compound previously tentatively identified as luteolin 7-*O*-glucoside has been confirmed to be luteolin 7-*O*-glucuronide, as described in Section 2.1. and Section 3.5. Due to the similar UV spectra and absorption maxima of the two compounds, the determination of luteolin 7-*O*-glucoside was equivalent to the quantitative determination of luteolin 7-*O*-glucuronide.

### 3.4. LC-PDA-ESI-MS Analysis of Satureja Extracts

The chromatographic separation was performed using a ZORBAX RRHD Eclipse XDB-C18 (150 × 2.1 mm, 1.8 µm) (Agilent, Santa Clara, CA, USA). The mobile phase was 0.2% (*v*/*v*) formic acid in ultra-pure water (A) and acetonitrile (B). The separation was achieved via a gradient of 0 min—0% B; 13.8 min—20% B; 16.2 min—22.5% B; 16.8 min—24.4% B; 18 min—26.2% B; 30 min—30% B; 36 min—100% B, which was followed by 7 min of equilibration. The injection volume was 1 µL, the flow rate was 0.22 mL/min, and the column temperature was maintained at 40 °C. The UV–Vis spectra were recorded from 190 to 540 nm with selective monitoring at 280 nm and 350 nm. The mass spectrometry (MS) parameters used for the ionization source were set as follows: MS/TOF: negative ionization; gas temperature: 300 °C; drying gas: 10 L/min; nebulizer: 50 psi; sheath gas temperature: 300 °C; sheath gas flow: 10 L/min; V Cap: 2500 V; nozzle voltage: 1000 V; fragmentor: 180 V; m/z range: 100–1100 m/z in 2 GHz Extended Dynamic Range mode. The data processing was performed using Mass Hunter Qualitative Analysis version 12.0 software.

### 3.5. Radical Scavenging Activity

The radical scavenging activity (RSA) was determined using DPPH free radicals [66]. A solution of 130 μL of the extract in methanol was mixed with a 70 μL, 0.21 mg/mL DPPH solution. After 30 min, the absorbance was recorded at 545 nm, and the RSA was calculated according to Equation (1):

(1) $$RSA\left(\%\right)=\frac{A_{0}-A_{s}}{A_{0}}\times 100$$

where A_0_ is the absorbance of the negative control, which used methanol instead of the extract, and A_s_ is the absorbance of the respective extract. The concentration of the extract that scavenged 50% of the free radicals present in the solution (RSA IC_50_) was calculated. BHA was used as the positive control.

### 3.6. Reducing Power

For the reducing power (RP) of the extracts [67], the extract solution (40 µL) was mixed with water (40 µL), phosphate buffer (0.2 M, pH 6.6, 100 µL), and potassium ferricyanide (1%, *w*/*w*, 100 µL). Trichloroacetic acid (10% *w*/*w*, 100 µL) was added to the mixture after 20 min of incubation at 50 °C. An amount of 25 µL of a 0.1% (*w*/*v*) ferric chloride solution was added to an aliquot of 125 µL of the supernatant. RP was measured as the absorbance at 700 nm As the RP increases linearly, it does not reach a maximum of 100% value, the concentration of the extract that achieved an absorbance of 0.5 at 700 nm (RP EC_0.5_) was calculated instead. ASC was used as the positive control.

### 3.7. Antioxidant Activity in the β-Carotene-Linoleic Acid Assay

For the determination of the antioxidant activity in the *β*-carotene-linoleic acid assay (ACL) [68], 200 μL of an emulsion containing 0.75 mg/mL *β*-carotene, 1.1 mg/mL linoleic acid, and 8.5 mg/mL Tween 40 was mixed with the extract solution in methanol (50 μL). The reaction mixture was incubated at 50 °C and the ACL was calculated based on the absorbances recorded at the beginning of the reaction and after 60 min using Equation (2):

(2) $$ACL\left(\%\right)=\frac{A_{s\left(t=60\right)}}{A_{c\left(t=0\right)}}\times 100$$

where A_c_ and A_s_ are the absorbances of the methanol control and extract, respectively. The concentration of the extract that protected 50% of the β-carotene present in the solution (ACL IC_50_) was calculated. BHA was used as the positive control.

### 3.8. Tyrosinase Inhibitory Activity

For the tyrosinase inhibitory activity (TyInh) [69], 80 μL of the extract solution and 40 μL of freshly prepared tyrosinase solution in the phosphate buffer (16 mM, pH 6.8) were mixed and incubated at room temperature for 10 min in the dark. Following this, 80 μL of l-DOPA solution (0.19 mg/mL in phosphate buffer) was added. The resulting absorbance was measured after 10 min at 492 nm, and the TyInh was calculated as in Equation (3):

(3) $$TyInh\left(\%\right)=\frac{A_{0}-A_{s}}{A_{0}}\times 100$$

where A_0_ is the absorbance of the negative control (where the buffer was used instead of the extract), and A_s_ is the absorbance of the respective extract. The concentration of the extract that inhibited 50% of the tyrosinase activity (TyInh IC_50_) was calculated. Kojic acid was used as the positive control.

### 3.9. Measurement of UV-A- and UV-B-Absorbing Capabilities of S. montana Extracts

The absorbance spectra of the extracts, diluted in water at a 1:64 (*v*/*v*) ratio, were recorded [70] across wavelengths ranging from 290 to 400 nm. The areas under the curves (AUCs) were quantified in two wavelength intervals: 290–320 nm (UV-B) and 320–400 nm (UV-A). A solution of PABA (1 mg/mL), diluted in the same manner as the extracts, served as the positive control.

### 3.10. Lipoxygenase Inhibitory Activity

For the LOX inhibitory activity (LOXInh) [71], 25 μL of the LOX solution (0.0032 mg/mL), 100 μL of the extract solution, and 50 μL of the phosphate buffer (pH 8.0, 100 mM) were mixed. After 5 min, 50 μL of linoleic acid in the phosphate buffer (pH 8.0, 100 μM) was mixed and incubated at 25 °C. After 45 min, the absorbance was measured at 234 nm. The LOX inhibitory activity (LOXInh) was calculated as in Equation (4):

(4) $$LOXInh\left(\%\right)=\frac{A_{0}-A_{s}}{A_{0}}\times 100$$

where A_0_ is the absorbance of the negative control (the reaction mixture containing the buffer solution instead of the extract), and A_s_ is the absorbance of the corresponding extract. The LOXInh IC_50_ was calculated as the concentration of the extract that inhibited 50% of the LOX activity and was expressed as μL of extract/mL of solution. NDGA was used as the positive control.

### 3.11. Inhibition of Heat-Induced Ovalbumin Coagulation

The heat-induced ovalbumin coagulation inhibition (OvInh) [53] was determined by mixing the extract (80 μL) and ovalbumin (90 μL) solution in phosphate-buffered saline (pH 7.4). The mixture was quickly mixed, incubated for 15 min at 37 °C, and then heated at 70 °C for 5 min. The intensity of the haze was estimated by recording the absorbance at 660 nm, and the OvInh was calculated using Equation (5):

(5) $$OvInh\left(\%\right)=\frac{A_{0}-A_{s}}{A_{0}}\times 100$$

where A_0_ is the absorbance of the negative control (water), and A_s_ is the absorbance of the respective extract. The concentration of the extract that inhibited 50% of the ovalbumin coagulation (OvInh IC_50_) was calculated. Diclofenac sodium was used as the positive control.

### 3.12. Cell Culture Conditions

The HaCaT cell line for the experiments was cultivated using DMEM supplemented with FBS, penicillin, streptomycin, and amphotericin B. The cells were passaged at 80–90% confluence, and the medium was changed approximately every 48 h. The cultures were maintained at 95% humidity and 37 °C in an atmosphere of 5% CO_2_.

### 3.13. Cell Viability Study

The cell viability was determined using the MTT test [72] and compared to untreated cells incubated in HBSS (the negative control). HaCaT cells were seeded onto the 96-well plates at a density of 2 × 10^4^ cells/well and incubated to reach confluence. After 24 h, the cell culture medium was removed and the cells were washed with HBSS. The extracts, previously diluted with HBSS, were used to treat the cells for 2 h. Thereafter, the cells were washed twice with HBSS and incubated with a fresh medium (100 µL/well). After 24 h, 20 µL of the MTT solution (2.5 mg/mL) was added to each well, and the plates were incubated at 37 °C. After 1 h, the medium was removed and the cells were lysed. The formazan formed in the reaction was dissolved with acidic isopropanol, and the absorbance at 570 nm was measured. The metabolic activity was expressed relative to the negative control.

### 3.14. In Vitro Scratch Wound-Healing Assay

HaCaT cells were cultured in 24-well plates at a density of 10^5^ cells per well [72], with a total volume of 500 µL per well. The cells were allowed to achieve sufficient confluence over a 24-h period in a DMEM medium enriched with 10% FBS and antibiotics. Following this incubation, the medium was discarded and substituted with a serum-free medium. After an additional 24 h, a sterile 10 µL pipette tip was employed to create a “wound” by scraping across the surface of each well, resulting in a cell-free area. The cell monolayer was subsequently washed gently with HBSS to eliminate any detached cells and debris. The wounds were then treated with extract solutions in HBSS for a duration of 2 h. Each well was marked beneath the plate to facilitate the identification of the same scratched area. After the 2-h treatment, the cells were washed again with HBSS and incubated with 500 µL of the serum-free medium per well. Wounds treated solely with HBSS served as the negative control. The in vitro epithelialization of the wounds was observed over 48-h period, with assessments made every 24 h using phase-contrast microscopy at a 10× magnification. The scratch area was quantified using ImageJ software 1.54c (National Institutes of Health, Bethesda, MD, USA), and the wound-healing rate (WHR) was calculated as the percentage of scratch closure relative to the initial scratch area, in accordance with Equation (6):

(6) $$WHR\left(\%\right)=\frac{A_{0}-A_{t}}{A_{0}}\times 100$$

where A_0_ is the scratch area at time 0, and A_t_ is the corresponding scratch area at 24 or 48 h.

### 3.15. Statistical Analysis

The antioxidant and enzyme-inhibiting activities are expressed as the mean ± standard deviation derived from three measurements. The IC_50_ and IC_0.5_ values were determined via regression analysis. Due to the nonvolatility of glycerol, the IC_50_ and IC_0.5_ values for the extracts were expressed as µL extract/mL. Conversely, the IC_50_ and IC_0.5_ values for the standards (solid substances) were expressed as µg/mL (equaling numeric value of volume equivalents of 1 mg/mL standard solution). For the wound-healing assay, two independent experiments were conducted, utilizing three wells for each treatment condition. Statistical analyses were performed using ANOVA (GraphPad Prism), followed by Tukey’s test for the comparisons between the extracts and Dunnett’s post-hoc test for comparison with the standard control. A *p*-value of less than 0.05 was deemed statistically significant.

## 4. Conclusions

In a continuation of previous efforts to prepare *S. montana* extracts suitable for direct use in cosmetic products, a comprehensive analysis of the chemical compositions and cosmeceutical properties of extracts prepared using HCDGUAE was conducted. The prepared extracts, rich in target compounds, demonstrated efficacy across all of the performed assays. Notably, their antiradical and anti-inflammatory properties were particularly significant, as evidenced by the low IC_50_ values in the DPPH radical scavenging, anti-lipoxygenase, and ovalbumin-induced coagulation assays. Furthermore, their biocompatibility as well as their capacity to absorb UV-A and UV-B radiation and accelerate wound healing assures their status as excellent candidates for further dermatological and cosmeceutical product development. With regard to the notable anti-inflammatory activity of the extracts, the investigation of the mechanism of action, such as the study of the influence of the extracts and/or their constituents on cytokines, might be one of the directions of future research. Western blot analyses of key markers (e.g., IL-6, TNF-α), in addition to the perspective in vivo validation experiments, could provide a valuable addition to the knowledge on the extracts’ mechanisms of the action, as well as the estimation of their potential to exert similar effects in clinical settings.

## Figures and Tables

**Table 1:** LC-MS and UV–Vis spectroscopic data for compounds observed in *S. montana* extracts.

No.	Rt (min)	UV Spectra λ max (nm)	Observed ^a^	Δ (ppm)	Formula	Fragmentation in Negative Ionization Mode	Predicted Compounds
1	14.532	270, 348	609.14343	−5.56	C_27_H_30_O_16_	369, 399, 489, **609**	luteolin *C*-dihexoside
2	15.036	272, 334	593.14913	−4.17	C_27_H_30_O_15_	353,383, 473, **593**	apigenin *C*-dihexoside
3	15.476	256, 266, **348**	637.10243	−3.21	C_27_H_26_O_18_	285, 351, **637**	luteolin *O*-diglucuronide
4	16.219	234, 282, **344**	477.06622	−2.83	C_21_H_18_O_13_	301, **477**	quercetin *O*-glucuronide
5	17.453	250, 268, 336	593.14785	−5.34	C_27_H_30_O_15_	179, 285, 593, 799	luteolin derivative
6	17.881	244, 340	843.15980	−3.15	C_38_H_36_O_22_	285, 463, 557, **843**	luteolin derivative
7	18.094	254, **348**	461.07185	−1.87	C_21_H_18_O_12_	285, **461**	**luteolin 7-*O*-glucuronide (s)**
8	20.148	252, 344	607.16478	−3.68	C_28_H_32_O_15_	299, 359,511, **607**	flavonoid *O*-deoxyhexosohexoside
9	20.835	232, 328	359.07699	−0.71	C_18_H_16_O_8_	197, **359**, 719	**rosmarinic acid (s)**
10	21.911	234, 324	537.10311	−1.58	C_27_H_22_O_12_	293, 493, **537**	lithospermic acid A isomer
11	24.296	288, 324	717.14371	−3.34	C3_6_H_30_O_16_	339, 493, **717**	salvianolic acid B isomer
12	24.692	236, 322	535.08748	−1.44	C_27_H_20_O_12_	177, 248, 359, **535**	sagecoumarin
13	25.293	234, 290, 322	493.11374	−0.71	C_26_H_22_O_10_	135, 295, **493**	salvianolic acid isomer

^a^ Exact mass of [M-H]^−^; bold: most abundant; s: reference substance.

**Table 2:** Conditions used for the preparation of the *S. montana* extracts [22].

Extracts	X_1_ (%, *w*/*w*)	X_2_ (mmol)	X_3_ (°C)	X_4_ (g)	X_5_ (min)	X_6_ (W)
OPT-TP	70	0	45	0.80	15	504
OPT-TPA-RA	70	0.15	20	0.77	9	288
OPT-TF	70	0.20	65	0.77	25	720
OPT-LG	57	0.34	20	0.80	14	288

X_1_—glycerol content; X_2_—(2-hydroxypropyl)-*β*-cyclodextrin amount; X_3_—temperature; X_4_—drug weight; X_5_—extraction time; X_6_—ultrasound power.

**Table 3:** Previously reported chemical compositions of the *S. montana* extracts [22].

Extracts	TP (μg/mL)	TPA (μg/mL)	TF (μg/mL)	RA (μg/mL)	LG (μg/mL)
OPT-TP	5936.7 ± 176.4	3294.8 ± 281.8	676.7 ± 46.6	44.5	50.8
OPT-TPA-RA	5487.7 ± 37.9	4172.2 ± 21.9	517.1 ± 46.1	1163.3	245.8
OPT-TF	4352.2 ± 141.4	3267.6 ± 112.2	991.2 ± 19	637.1	103.7
OPT-LG	3663.8 ± 113.8	2708.7 ± 151.5	479.6 ± 45.9	930.6	284.0

TP—total phenolic content; TPA—total phenolic acid content; TF—total flavonoids; RA—rosmarinic acid; LG—luteolin 7-*O*-glucuronide (previously tentatively identified as luteolin 7-*O*-glucoside).

## Data Availability

Data are contained within the article and supplementary materials.

## Supplementary Materials

The following supporting information can be downloaded at: https://www.mdpi.com/article/10.3390/molecules30122638/s1, Table S1. The influence of the *S. montana* extracts (prepared as described in Table 2) in different concentrations on the HaCaT cell viability compared to cells treated with HBSS (designated to have 100% viability). ^a,b^ Differences between the extracts within the same concentration (within the same row) (ANOVA followed by Tukey’s post-test, *p* < 0.05). Figure S1. The UV chromatograms of the *S. montana* extracts. The compound labels are explained in Table 1 (the main text) while the extracts were prepared as described in Table 2 in the main text. Figure S2. The MS spectrum of compound **1** predicted as luteolin *C*-dihexoside. Figure S3. The MS spectrum of compound **2** predicted as apigenin *C*-dihexoside. Figure S4. The MS spectrum of compound **3** predicted as luteolin O-diglucuronide. Figure S5. The MS spectrum of compound **4** predicted as quercetin *O*-glucuronide. Figure S6. The MS spectrum of compound **5** predicted as luteolin derivative. Figure S7. The MS spectrum of compound **6** predicted as luteolin derivative. Figure S8. The MS spectrum of compound **7** predicted as luteolin 7-*O*-glucuronide. Figure S9. The MS spectrum of compound **8** predicted as flavonoid *O*-deoxyhexosohexoside. Figure S10. The MS spectrum of compound **9** predicted as rosmarinic acid. Figure S11. The MS spectrum of compound **10** predicted as lithospermic acid A isomer. Figure S12. The MS spectrum of compound **11** predicted as salvianolic acid B isomer. Figure S13. The MS spectrum of compound **12** predicted as sagecoumarin. Figure S14. The MS spectrum of compound **13** predicted as salvianolic acid isomer. Figure S15. The influence of the OPT-TP (a–c), OPT-TPA-RA (e–g), OPT-TF (h–j), OPT-LG (k–m), and HBSS (n–p), in 31.3 extract/mL dilutions on the closure of scratch in HaCaT cells monolayer after (a,e,h,k,n), 24 h (b,f,i,l,o) and 48 h (c,g,j,m,p) after being incubated with the extracts or HBSS for 2 h.

## Abbreviations

The following abbreviations are used in this manuscript:

ACL	antioxidant activity in the β-carotene-linoleic acid assay
ASC	ascorbic acid
AUC	areas under the curve
BHA	butylated hydroxyanisole
CD	cyclodextrin
COX	cyclooxygenase
DMEM	dulbecco’s modified Eagle’s medium
DPPH	2,2-diphenyl-1-picrylhydrazyl
FA	formic acid
GSH-Px	glutathione peroxidase
HaCaT	human aneuploid immortal keratinocyte cell line
HBSS	hank’s balanced salt solution
HCDGUAE	hydroxypropyl-*β*-cyclodextrin-glycerol ultrasound-assisted extraction
HP-*β*-CD	hydroxypropyl-*β*-cyclodextrin
IL	interleukin
L-DOPA	3,4-dihydroxyphenylalanine
LOX	lipoxygenase
LOXInh	lipoxygenase inhibitory activity
MMP	metalloproteinase
MTT	3-(4,5-dimethylthiazol-2-yl)-2,5-diphenyltetrazolium bromide
NDGA	nordihydroguaiaretic acid
NF-κB	nuclear factor k-light-chain-enhancer of activated B cells
NRF2	nuclear factor erythroid 2-related factor
OPT-LG	*S. montana* extract rich in luteolin derivatives
OPT-TF	*S. montana* extract rich in total flavonoids
OPT-TP	*S. montana* extract rich in total phenols
OPT-TPA-RA	*S. montana* extract rich in total phenolic acids, including rosmarinic acid
OvInh	heat-induced ovalbumin coagulation inhibition
PABA	*p*-aminobenzoic acid
RP	reducing power
RSA	radical scavenging activity
SOD	superoxide dismutase and induced by
TLR	toll-like receptor
TNF-α	tumor necrosis factor alpha
TyInh	tyrosinase inhibitory activity
UV	ultraviolet
WHR	wound-healing rate
