# Supplementary material for: Cosmeceutical and Wound-Healing Activities of Green Hydroxypropyl-β-Cyclodextrin-Glycerol-Based Satureja montana Extracts
Source: Molecules. 2025 Jun 18;30(12):2638. doi: 10.3390/molecules30122638 (PMC12195765; doi:10.3390/molecules30122638)
Supplement: Supplementary file 1 [file molecules-30-02638-s001.zip › molecules-3664639-supplementary.pdf]

Supplementary Material

Cosmeceutical and Wound-Healing Activities of Green Hydroxypropyl-β-Cyclodextrin-Glycerol-Based *Satureja montana* Extracts

Lejsa Jakupović[1], Jakub W. Strawa[2], Laura Nižić Nodilo[3], Marijan Marijan[1], Anita Hafner[3], Katarzyna Jakimiuk[2], Monika Tomczykowa[4], Michał Tomczyk[2] and Marijana Zovko Končić[1,*]

[1] Department of Pharmacognosy, University of Zagreb Faculty of Pharmacy and Biochemistry, A. Kovačića 1, 10000 Zagreb, Croatia
[2] Department of Pharmacognosy, Faculty of Pharmacy with the Division of Laboratory Medicine, Medical University of Białystok, ul. Mickiewicza 2a, 15-230 Białystok, Poland
[3] Department of Pharmaceutical Technology, University of Zagreb Faculty of Pharmacy and Biochemistry, A. Kovačića 1, 10000 Zagreb, Croatia
[4] Department of Organic Chemistry, Faculty of Medicine with the Division of Dentistry and Division of Medical Education in English, Medical University of Białystok, ul. Mickiewicza 2a, 15-222 Białystok, Poland

* Correspondence: marijana.zovko@pharma.unizg.hr

Table S1. The influence of the *S. montana* extracts (prepared as described in Table 2) in different concentrations on the HaCaT cell viability compared to cells treated with HBSS (designated to have 100% viability). [a,b] Differences between the extracts within the same concentration (within the same row) (ANOVA followed by Tukey's post-test, $p < 0.05$).

Concentration	Viablilty (%)			
(µL extract/mL)	OPT-TP	OPT-TPA-RA	OPT-TF	OPT-LG
7.8	99.6 ± 10.6[a]	116.9 ± 15.1[a]	112.3 ± 4.0[a]	104.9 ± 6.8[a]
15.7	95.9 ± 5.3[a]	115.9 ± 13.9[a]	112.5 ± 4.8[a]	100.6 ± 2.9[a]
31.3	104.9 ± 8.6[a]	117.4 ± 13.4[a]	111.0 ± 6.8[a]	109.0 ± 8.0[a]
62.5	103.4 ± 8.6[a]	108.9 ± 11.0[a]	106.3 ± 9.5[a]	94.0 ± 4.3[a]
125	81.5 ± 7.4[a]	61.3 ± 2.0[b]	93.8 ± 4.3[a]	64.6 ± 3.2[b]
250	45.6 ± 1.2[a]	40.8 ± 4.2[a]	37.4 ± 6.6[a]	34.8 ± 2.6[a]

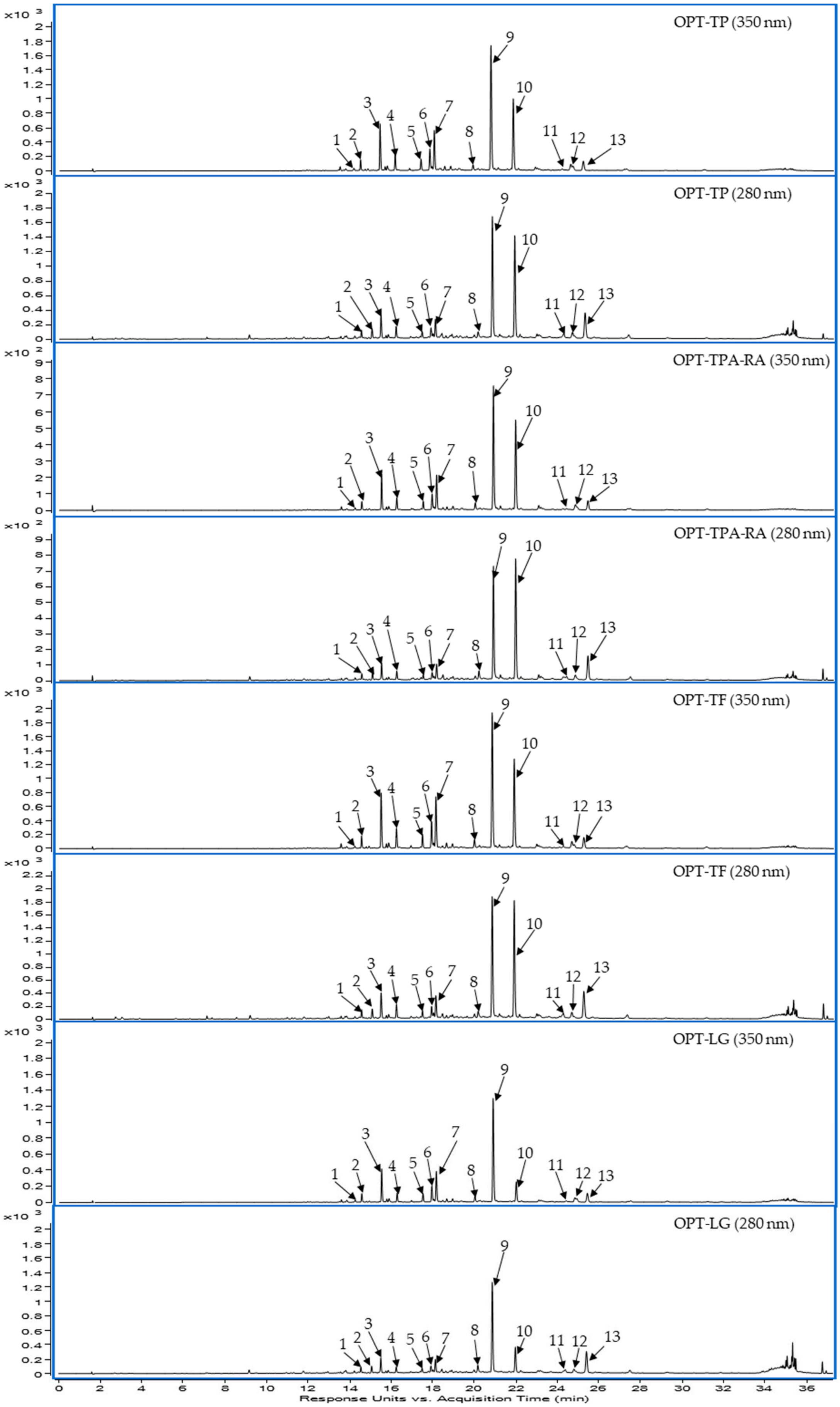

Figure S1. The UV chromatograms of the *S. montana* extracts. The compound labels are explained in Table 1 (the main text) while the extracts were prepared as described in Table 2 in the main text.

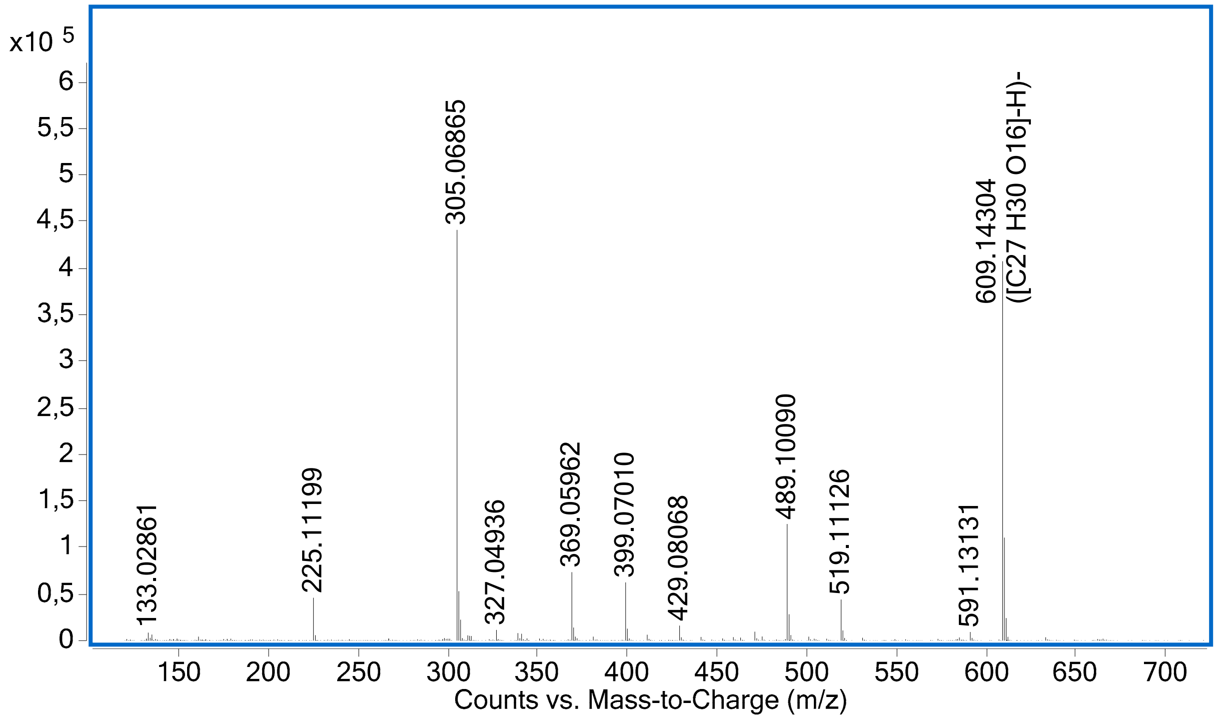

Figure S2. The MS spectrum of compound 1 predicted as luteolin *C*-dihexoside.

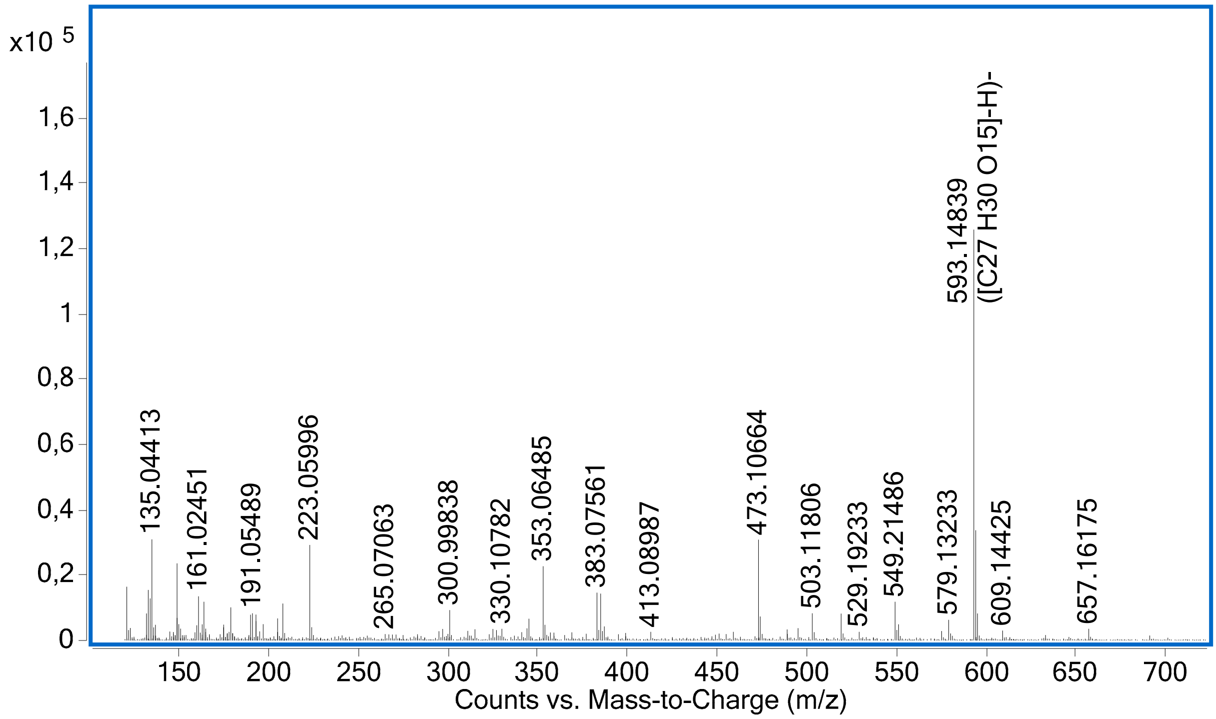

Figure S3. The MS spectrum of compound 2 predicted as apigenin *C*-dihexoside.

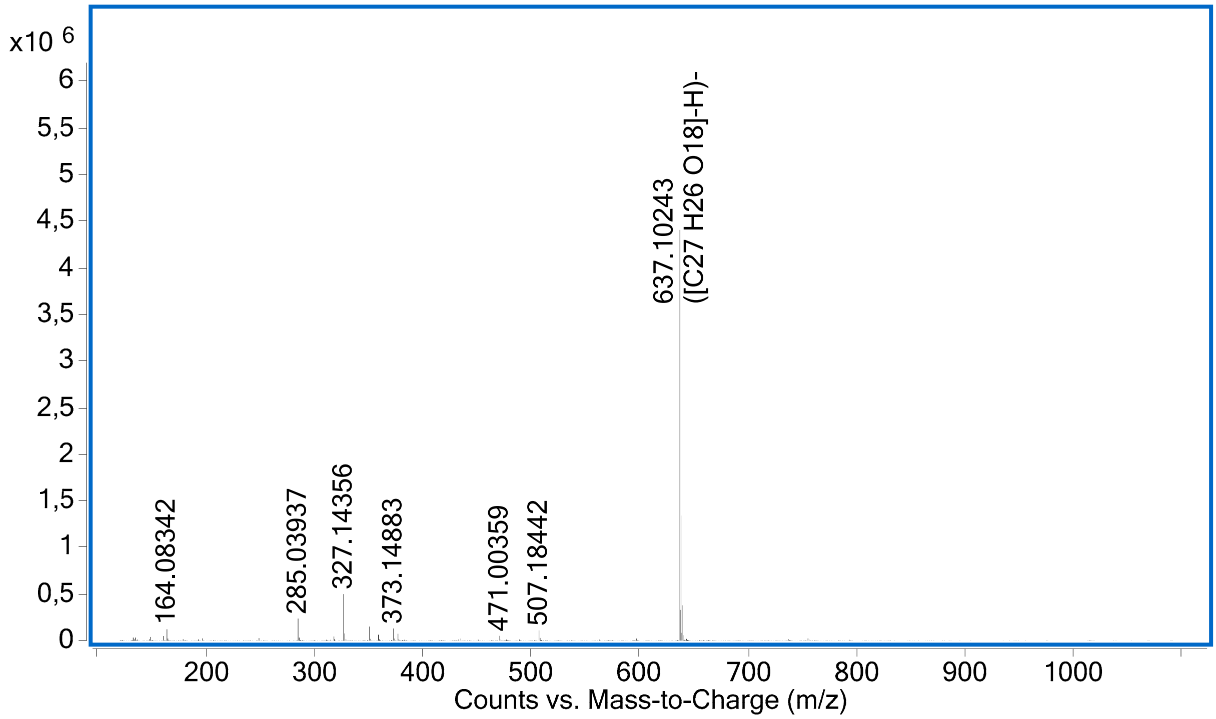

Figure S4. The MS spectrum of compound 3 predicted as luteolin *O*-diglucuronide.

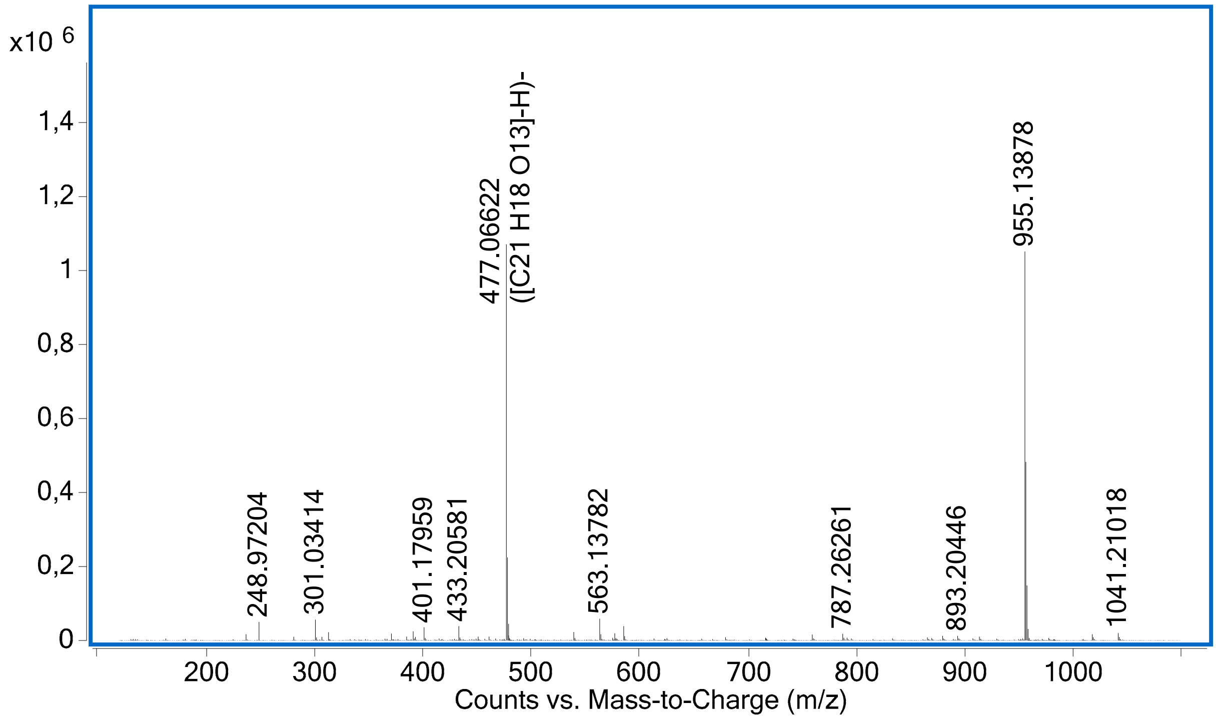

Figure S5. The MS spectrum of compound 4 predicted as quercetin *O*-glucuronide.

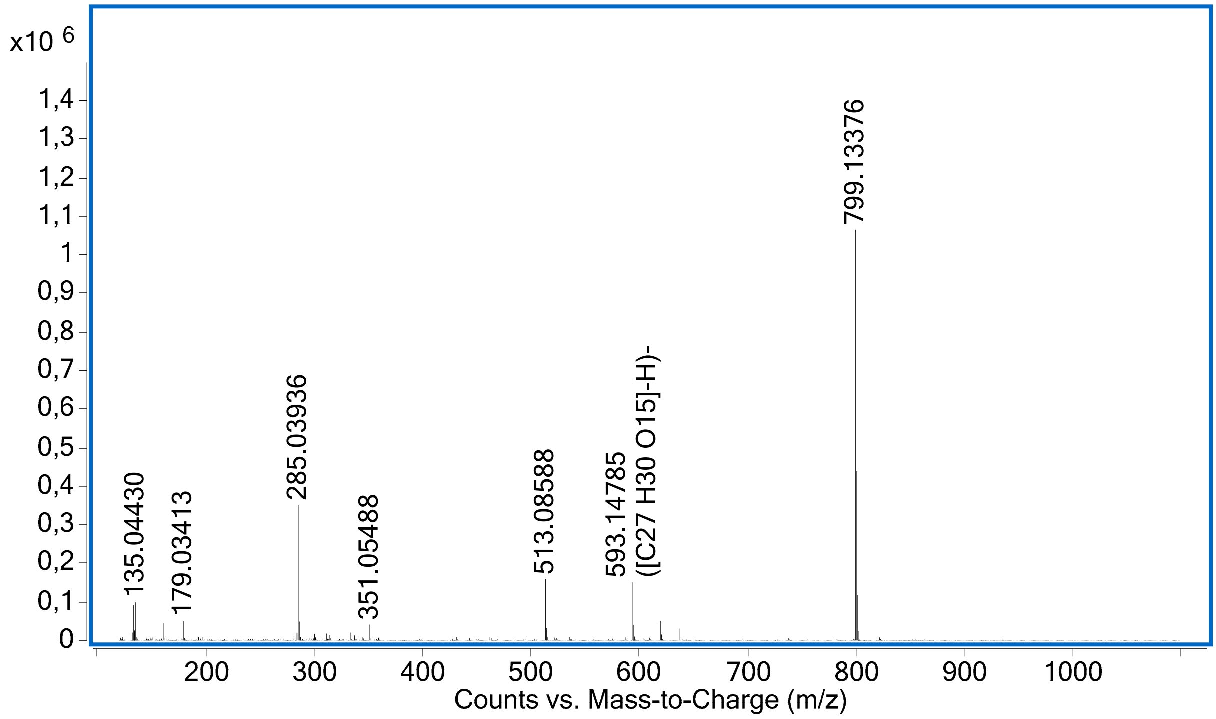

Figure S6. The MS spectrum of compound 5 predicted as luteolin derivative.

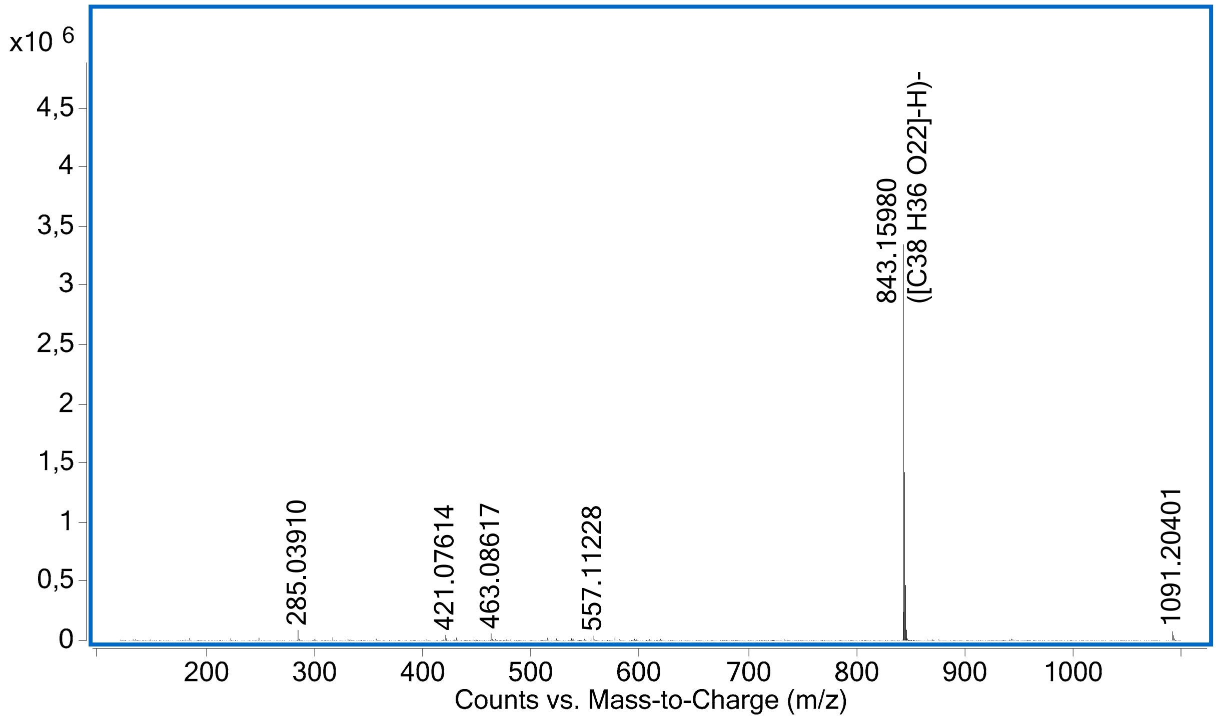

Figure S7. The MS spectrum of compound 6 predicted as luteolin derivative.

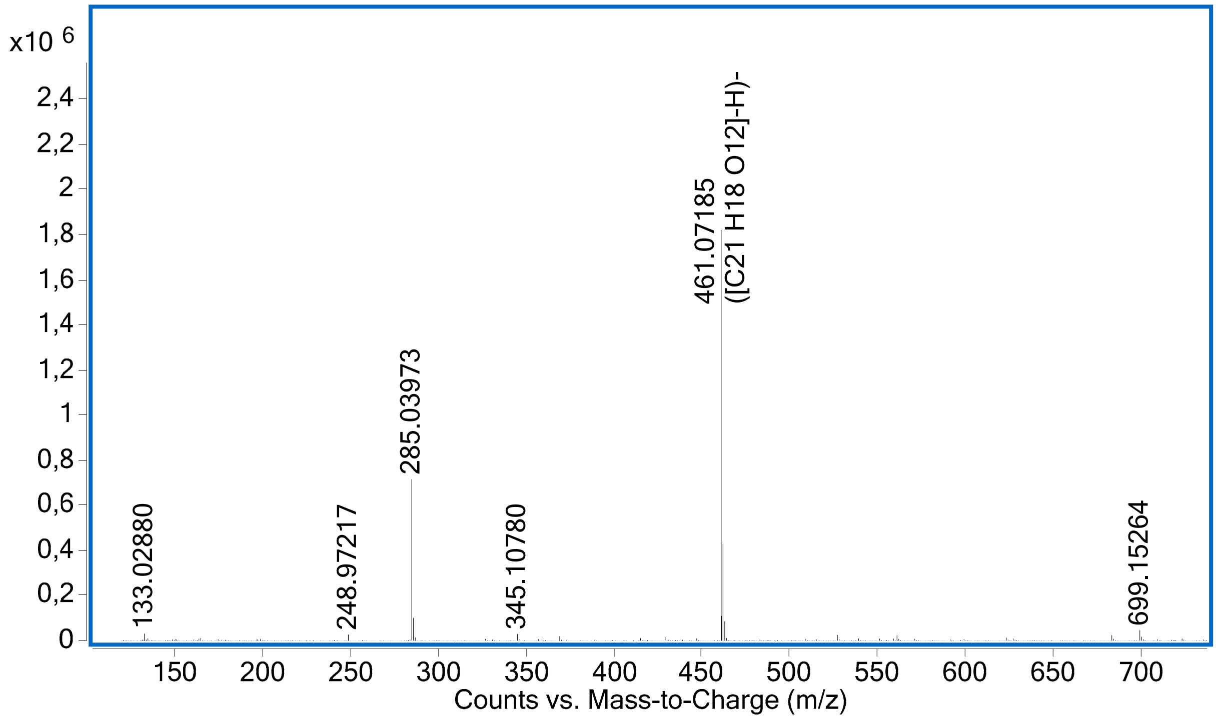

Figure S8. The MS spectrum of compound 7 predicted as luteolin 7-*O*-glucuronide.

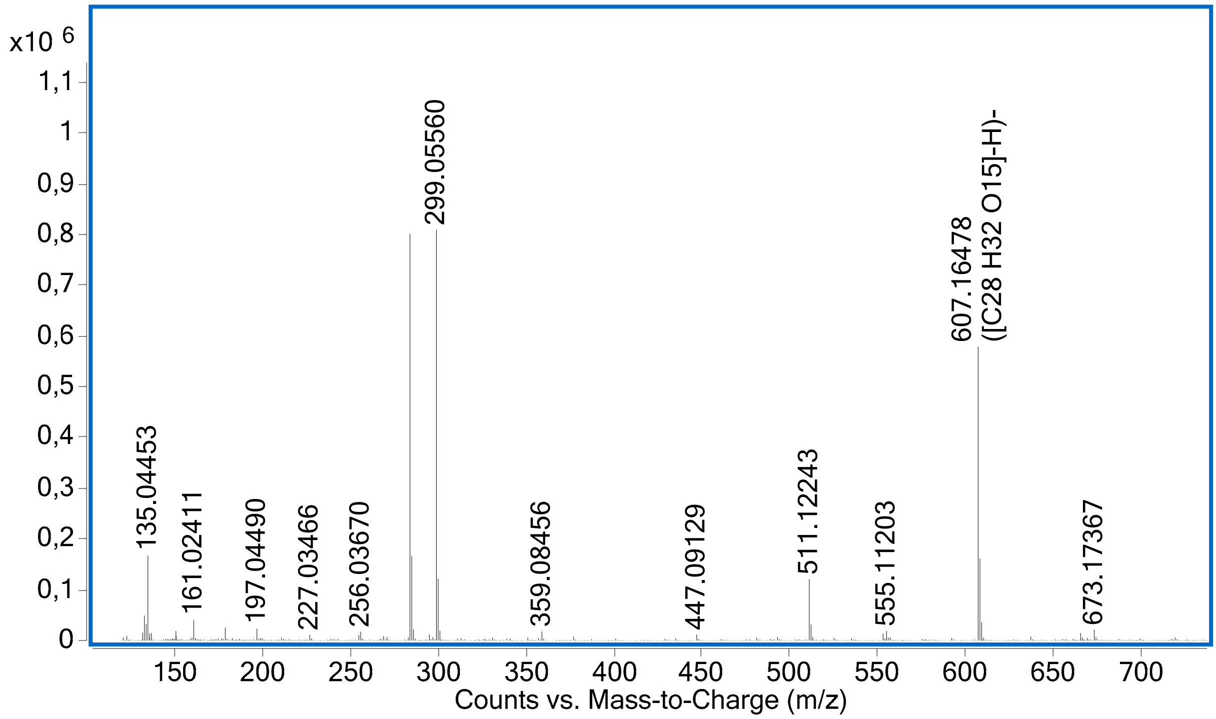

Figure S9. The MS spectrum of compound 8 predicted as flavonoid *O*-deoxyhexosohexoside.

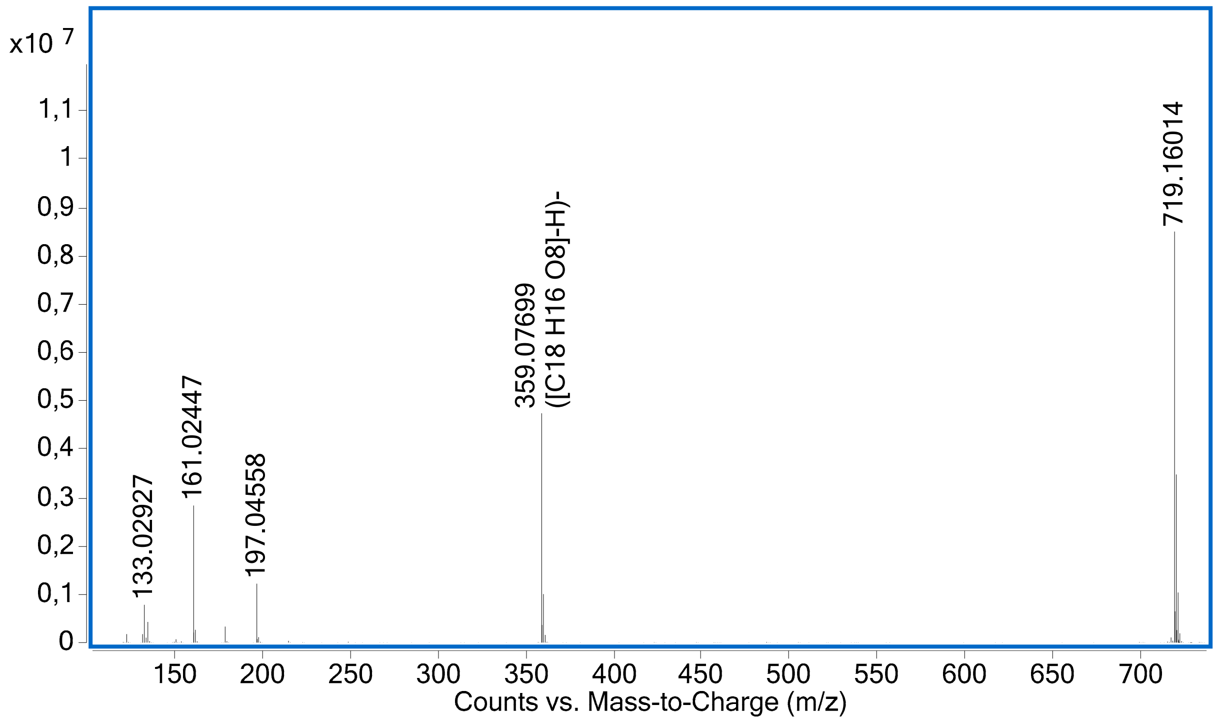

Figure S10. The MS spectrum of compound 9 predicted as rosmarinic acid.

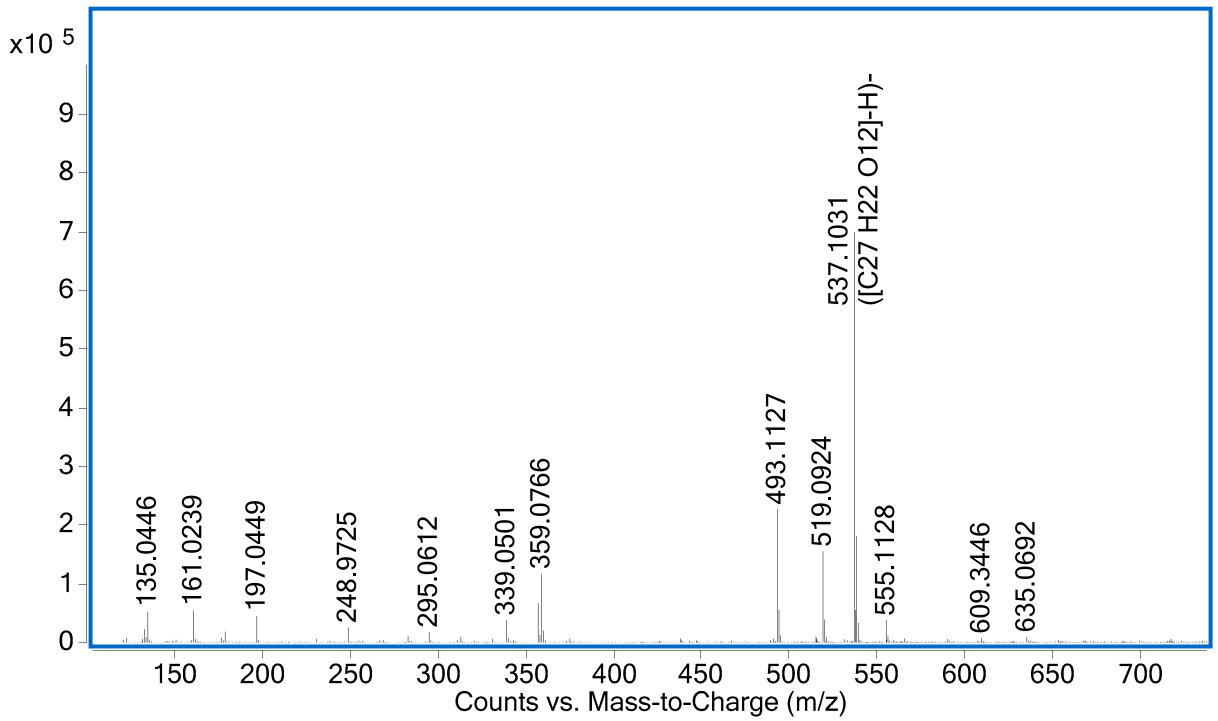

Figure S11. The MS spectrum of compound 10 predicted as lithospermic acid A isomer.

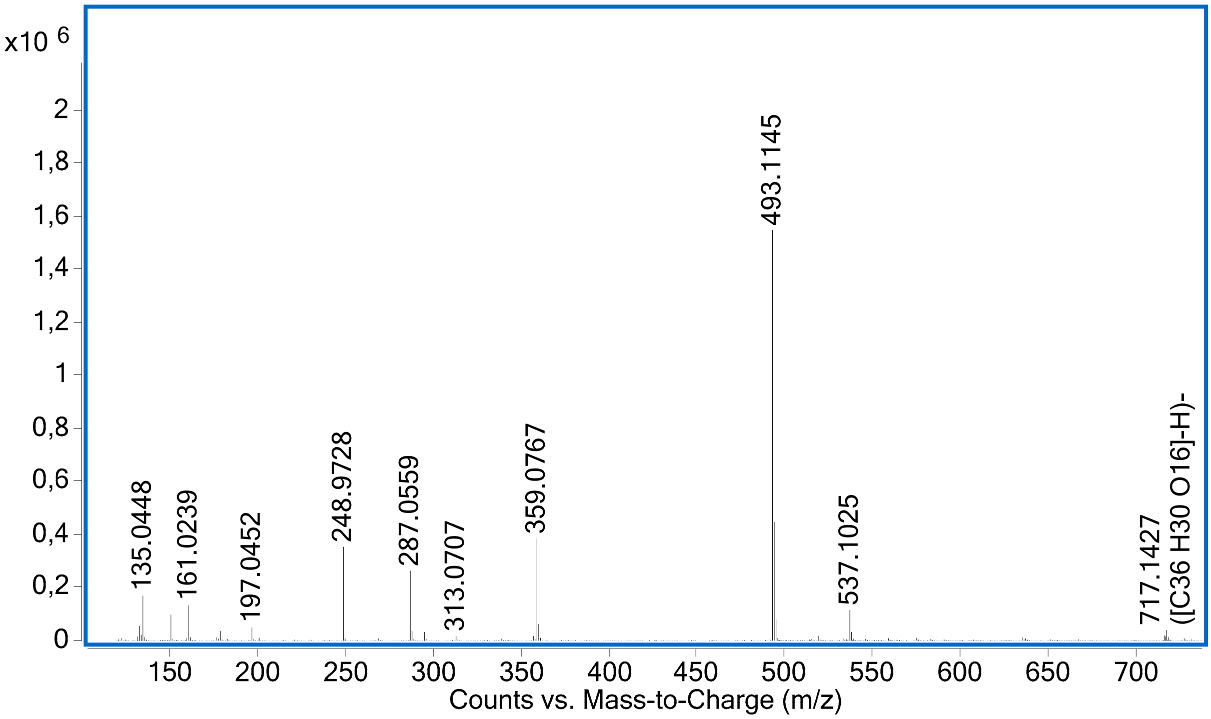

Figure S12. The MS spectrum of compound 11 predicted as salvianolic acid B isomer.

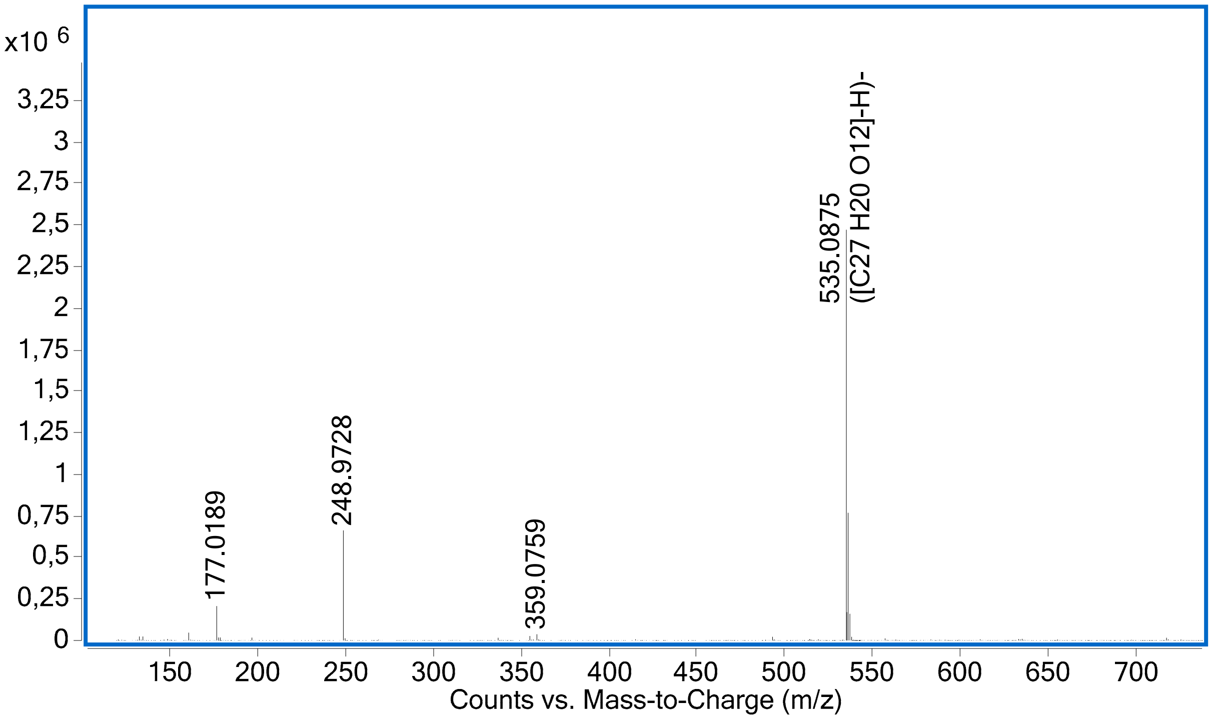

Figure S13. The MS spectrum of compound 12 predicted as sagecoumarin.

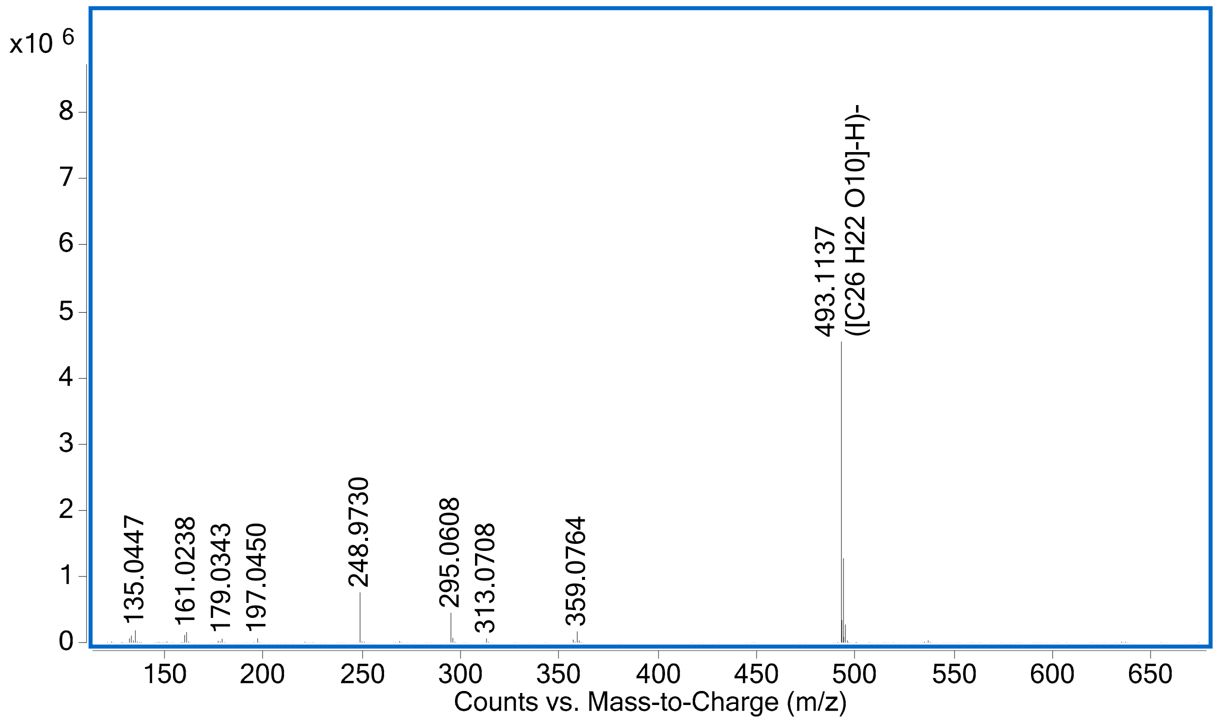

Figure S14. The MS spectrum of compound 13 predicted as salvianolic acid isomer.

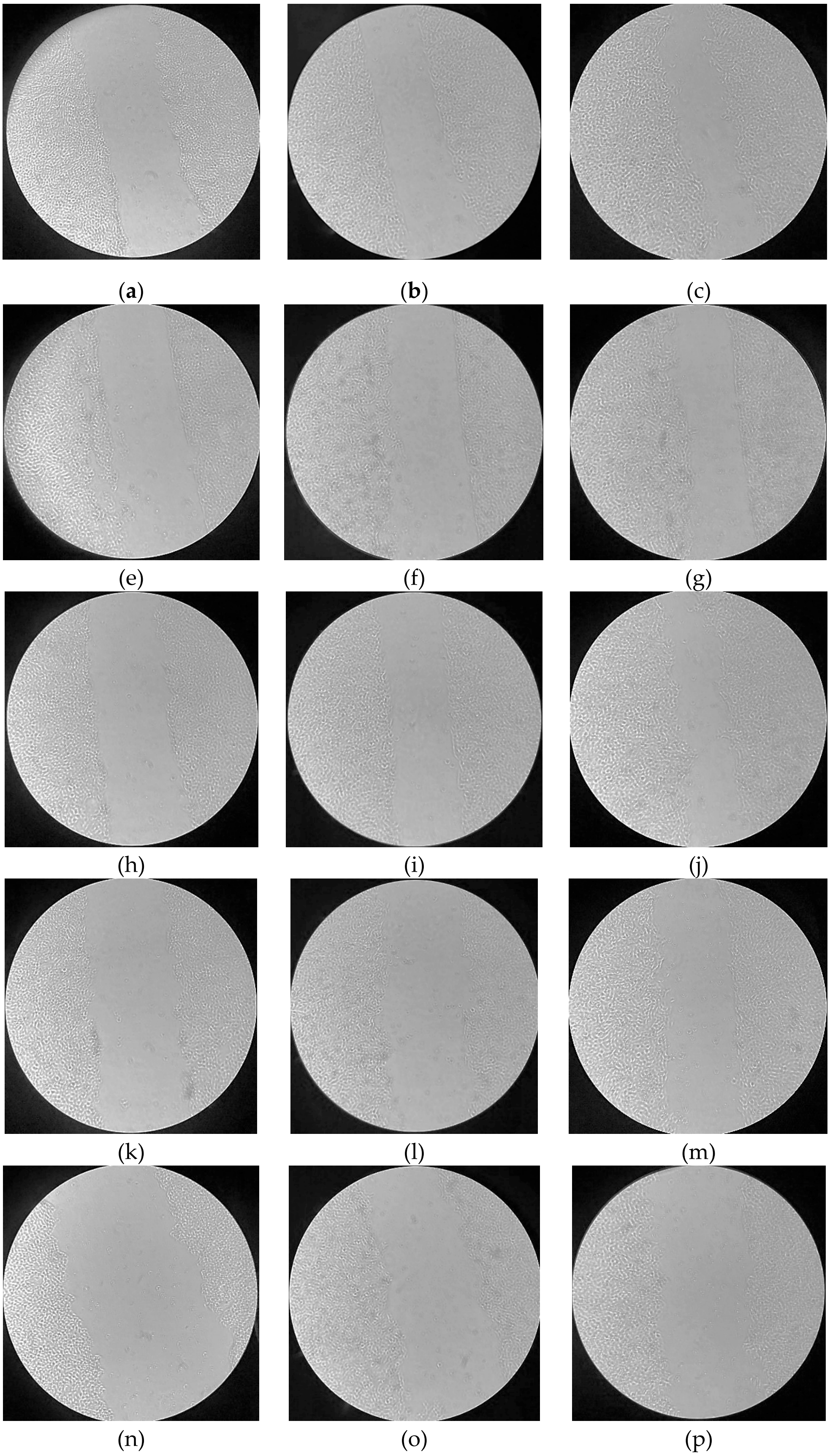

Figure S15. The influence of the OPT-TP (a-c), OPT-TPA-RA (e-g), OPT-TF (h-j), OPT-LG (k-m), and HBSS (n-p), in 31.3 extract/mL dilutions on the closure of scratch in HaCaT cells monolayer after 0h (a,e,h,k,n), 24h (b,f,i,l,o) and 48h (c,g,j,m,p) after being incubated with the extracts or HBSS for 2 h.
